# Correlation of MRI Viability With Grade of Collaterals in Coronary Arteries With Chronic Total Occlusions

**DOI:** 10.1016/j.jscai.2025.102573

**Published:** 2025-04-01

**Authors:** Esad Vucic, Danielle Retcho, Sumit Sohal, Kyrillos Girgis, Dorothy Amofa, Avni Garg, Aanchal Wats, Brian Wosnitzer, Marc Cohen, Sergio Waxman, Najam Wasty

**Affiliations:** a Section of Cardiovascular Medicine, Department of Internal Medicine, Newark Beth Israel Medical Center, Newark, New Jersey; b Section of Cardiovascular Medicine, Department of Internal Medicine, University of Pittsburgh Medical Center, Altoona, Pennsylvania; c Department of Radiology, Newark Beth Israel Medical Center, Newark, New Jersey

**Keywords:** cardiac MRI, chronic total occlusion, collaterals, viability

## Introduction

Myocardial cells undergo metabolic adaptations and biochemical dysfunction depending upon the severity and duration of ischemia. While in setting of severe and prolonged myocardial ischemia, cells may die, but in states of transient or chronic low flow states, these cells may stay alive and viable.^1^ Cardiac magnetic resonance imaging (CMR) with late gadolinium enhancement (LGE) can help to ascertain cardiac viability by detecting regions of myocardial fibrosis, a marker of nonviable myocardium as areas of high intensity signal in contrast to the surrounding nulled myocardium.^2^ These viable cells need essentials for resting metabolism which may be provided by collaterals in patients with chronic total occlusions (CTOs) of coronary arteries.^3^ The robustness of these collaterals may be related to the degree of viability in the areas meant to be supplied by CTO vessels. Therefore, in this article, we seek to compare and correlate the robustness of collaterals assessed by Rentrop classification^4^ to the degree of viability elicited by CMR, following which we discuss the implications of this finding.

## Methods

In this retrospective analysis, we reviewed all CMR studies performed at our institution between January 2019 and June 2023. The patients were included if they had a coronary angiogram that was performed within 14 days of the CMR. Patients with a history of coronary artery bypass grafts, remote angiograms, end-stage renal disease, or advanced chronic kidney disease (stage IV or above) were excluded. Only left anterior descending (LAD) and dominant right coronary artery (RCA) CTOs were evaluated in this study. The overall territory supplied was labeled as viable if >50% of the segments supplied by LAD/RCA had <50% LGE on CMR. The degree of collaterals were assessed by Rentrop classification from grade 0 being no collaterals; grade 1, filling of side branches of the artery without filling of main epicardial vessel; grade 2, partial filling of the main epicardial vessels; to grade 3, with complete filling of the main epicardial vessel.^4^ Two coronary angiographers (S.S. and N.W.) independently reviewed the studies and graded the collaterals. Thereafter, the grade of the collaterals on angiogram was correlated with the CMR-derived viability as determined by experienced imager with special expertise in CMR. The CMR reader was blinded to the degree of collaterals and coronary angiogram. The study was approved by the institutional review board.

## Results

**Figure 1:**
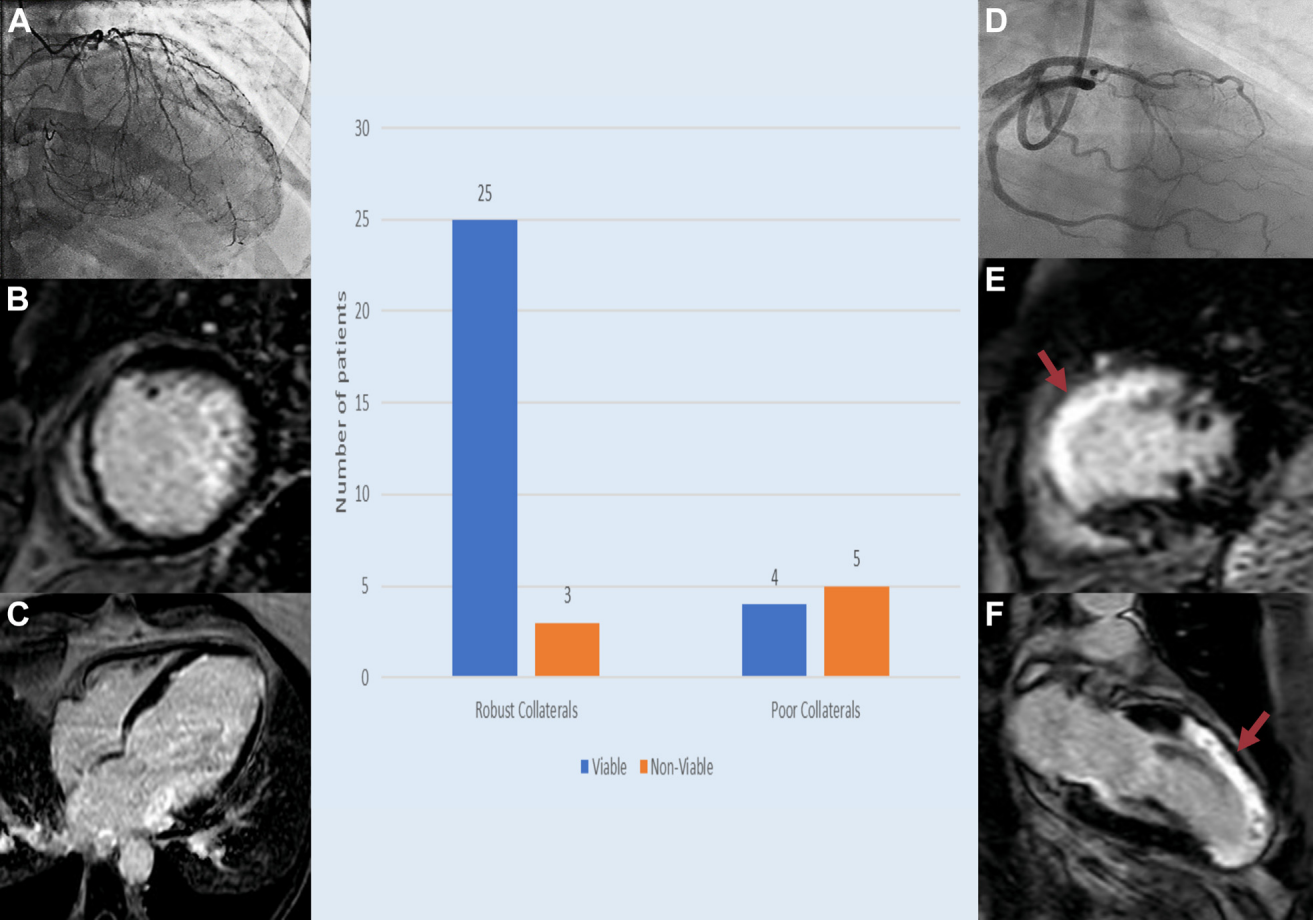
**Graphical presentation of degree of angiographic collaterals to the cardiac magnetic resonance imaging (MRI)-derived viability of the myocardium in patients with chronic total occlusion.** (A) Robust collaterals of RCA and LAD with concomitant viability with <50% late gadolinium enhancement in the respective territories on cardiac MRI in short axis (B) and long axis (C). (D) Poor collaterals of LAD with concomitant nonviability with >50% late gadolinium enhancement (red arrows) in the LAD territories on cardiac MRI in short axis (E) and long axis (F) Created in BioRender. S. Sohal (2025). https://BioRender.com/s95k830.

Of the 62 angiograms reviewed, 40 had LAD or RCA CTOs (24 and 16, respectively). The mean age of these patients was 58.6 years with 29 male and 11 female patients; 37% of the patients had diabetes and 40% had hypertension. All patients had reduced ejection fraction. Three patients were further excluded due to suboptimal LGE CMR studies. Of the final 37 patients, 28 had robust collaterals (grade 2 or 3) whereas 9 had poor (grade 0 or 1) collaterals. Moreover, 89.29% of the patients with robust collaterals showed viability on CMR compared with 44.44% of patients with poor collaterals (Figure 1). All patients with any viability by LGE CMR had at least some collaterals (Rentrop grade 1-3). Presence of robust collaterals had a sensitivity of 86.21% and specificity of 62.5% of finding viable tissue with a positive predictive value of 89.29% and negative predictive value of 55.55%.

## Discussion

In this analysis, we found that presence of robust collaterals filling the epicardial CTO vessel either partially or fully had a high sensitivity (86.21%) and positive predictive value (89.29%) of finding viability in the segment vascularized by that vessel but absence of these collaterals had a lower negative predictive value (55.56%). A corollary of this observation would be that the presence of robust collaterals connotes viable myocardium, but their absence may not preclude viability. Despite the use of variable definitions for viability and collateral grading, our results are in concordance with the similar studies done worldwide. Schumacher et al^5^ in their analysis also found an association of less myocardial scar with well-developed collaterals whereas poorly developed collaterals did not explicitly exclude viability. Similarly, Ripley et al^6^ also demonstrated association of good contralateral collateralization with myocardial viability.

Viable myocardium in an environment of decreased blood supply (such as CTO) needs nutrients and oxygen to maintain its metabolic and contractile functions and thus may promote more angiogenesis through various proangiogenesis factors leading to more collaterals; however, a scar tissue with no viable tissue may not promote the production of new blood vessels.^7^^,^^8^ An additional and complementary explanation would be that individual differences exist on how facile collateral vessels can be recruited, thereby determining the viability in a territory that is affected by the CTO.^4^ The resulting improved blood flow may further keep the tissue alive and thus maintaining its viability. This process may explain the findings of our study and such findings may facilitate a multidisciplinary discussion about the need for further confirmative testing for myocardial viability in patients with CTOs and robust collaterals. Certain limitations such as a retrospective, observational nature of our study, small sample size and subjective interpretation of Rentrop classification may lead to bias in our study, and hence, larger studies are needed to further corroborate our findings.
